# The Potential Role of the Microbiome in the Pathogenesis of Nasal Tumors: A Comprehensive Review

**DOI:** 10.3390/medicina60111808

**Published:** 2024-11-04

**Authors:** Antonella Loperfido, Davide Rizzo, Bruno Fionda, Luca Mureddu, Andrea Tondo, Luca Tagliaferri, Gianluca Bellocchi, Giovanni Delogu, Francesco Bussu

**Affiliations:** 1 Otolaryngology Unit, San Camillo Forlanini Hospital, 00152 Rome, Italy; aloperfido@scamilloforlanini.rm.it (A.L.);; 2 Otolaryngology Division, Azienda Ospedaliera Universitaria di Sassari, 07100 Sassari, Italy; 3 Department of Medicine Surgery and Pharmacy, Sassari University, 07100 Sassari, Italy; 4 UOC Degenze di Radioterapia Oncologica, Dipartimento di Diagnostica per Immagini e Radioterapia Oncologica, Fondazione Policlinico Universitario A. Gemelli IRCCS, 00168 Rome, Italy; 5 Istituto di Radiologia, Università Cattolica del Sacro Cuore, 00168 Rome, Italy; 6 Dipartimento di Scienze Biotecnologiche di Base, Cliniche Intensivologiche e Perioperatorie-Sezione di Microbiologia, Università Cattolica del Sacro Cuore, 00168 Rome, Italy; 7 Mater Olbia Hospital, 07026 Olbia, Italy

**Keywords:** nasal tumors, squamous cell carcinoma, SCC, nasal vestibule, paranasal sinuses, microbiome, dysbiosis, Human Papilloma Virus, HPV, carcinogenesis

## Abstract

Cancers of the nose, and especially the nose vestibule, represent a significant challenge for clinicians due to their rarity, the intricate nature of surrounding vital structures, the nonspecific early symptoms, and the etiological factors that are not completely understood. Emerging research suggests that alterations in the nasal microbiome, also known as microbial dysbiosis, may contribute to the pathogenesis of those malignancies through mechanisms involving chronic inflammation, immune modulation, and cellular changes. The aims of this paper are to review the current literature covering the nasal microbiome’s role in carcinogenesis, particularly in the context of squamous cell carcinoma, and to explore how microbial dysbiosis might foster a pro-tumorigenic environment. It further discusses potential future directions for research and therapeutic approaches.

## 1. Introduction

Primary malignancies of the nasal cavity and paranasal sinuses are rare, accounting for less than 3% of cancers of the upper aerodigestive tract, and less than 0.5% of all cancers [1]. They pose a significant challenge for clinicians, not only due to their rarity but also because of the nonspecific early symptoms, the great variety of histological types, and the complex anatomy, which, depending on the different subsites, deeply influences patterns of spread and ultimately the therapeutic approach and prognosis. Moreover, the main potential causes and risk factors are not completely understood. Various malignant neoplasms can develop in the nasal cavity and paranasal sinuses, with the most common being squamous cell carcinoma (SCC) and adenocarcinoma variants, followed by neuroendocrine tumors and mucosal melanoma. Sinonasal SCC encompasses a broad range of tumors with diverse biological characteristics, as indicated by its genetic similarities to other sinonasal cancers, including sinonasal undifferentiated carcinoma (SNUC) and neuroendocrine carcinoma (NEC). Of these tumors, SCC, although not as predominant as in the other head and neck sites, is probably still the most frequent type identified on histology [2]. In the detection of SCC of the nose, it is essential to specify and distinguish between those affecting the nasal cavities and paranasal sinuses and those originating from the nasal vestibule. The latter have recently been a focus of interest, as the need for the standardization of definition, classification, and treatment approach has clearly emerged. At present, the AJCC staging criteria for the primary lesion (T) in the nasal vestibule are the same as those for the nasal cavity proper and the ethmoid sinus [3,4,5]. Therefore, the current shared international staging system does not take into account the anatomical features of these different regions and the particular spread pattern of the different tumors. According to the literature, SCC of the nasal vestibule is also considered rare among the nasal and paranasal malignancies, with an estimated annual incidence of 0.41 cases per 100,000 people and accounts for approximately 1% of all head and neck cancers. However, this prevalence might be greatly underestimated because no specific topographic WHO code exists that allows for proper retrieval from the cancer registries, and also because of the common misdiagnosis with skin primaries [6]. This can lead to deferred treatment and a consequent deterioration of survival rates [7,8].

The definition of clear radiological anatomic boundaries, and consequently a specific WHO topographic code, is a prerequisite to allow proper assessment of the real incidence. Clinicians’ awareness of the possibility of a vestibule malignancy primary diagnosis should lead to recommending an endoscopic evaluation to exclude an origin inside the nasal vestibule in all SCCs arising around the nostril [9].

The incidence of nasal vestibule SCC peaks in the seventh decade of life, with a mean age between 67 and 71 years, and with a slightly higher occurrence in men. Typically, patients may refer to pain in the nasal vestibule, nasal obstruction, irritative symptoms like a burning sensation in the nose, and bleeding. Regarding risk factors, several trigger agents have been proposed including smoking, sunlight exposure, blackboard chalk typically used by teachers, and viral agents such as Human Papilloma Virus (HPV) and Epstein–Barr Virus (EBV), but the precise role of each remains contested [10].

Non-vestibular sinonasal SCC (mainly arising from the maxillary sinus, nasal cavity proper, and ethmoid) is a rare and aggressive malignancy arising from the epithelial cells lining the nasal cavity and paranasal sinuses. It has an annual incidence of about 1 in 100,000 people in most developed countries, accounting for less than 1% of all cancers and less than 4% of those originating in the head and neck area [11]. The low absolute risk in the general population has been associated with a high relative risk for specific chemical exposures and occupational environments, including woodworking, exposure to leather dust, formaldehyde, glues, nickel, chrome, arsenic and welding fumes, the textile industry, hairdressers and rubber workers [12,13]. Similarly to nasal vestibule SCC, this entity primarily affects men aged 50–70, with a male-to-female ratio of 2:1 [14]. Although environmental exposures, including wood and leather dust, tobacco smoke, HPV, and certain occupational hazards, have been identified as risk factors, the evidence on the matter is inconclusive, and the pathogenesis of sinonasal SCC remains largely unclear [15]. Another phenomenon described in the literature is the association between sinonasal SCC and inverted sinonasal papilloma [16]. Moreover, as reported for nasal vestibule SCC, this malignant entity is difficult to diagnose in the early stages, mainly due to its anatomical location and late-stage appearance with vague initial symptoms such as nasal obstruction, chronic nasal secretion, epistaxis, and facial pain [17]. In these anatomical regions, accurately determining the tumor’s site of origin can be challenging, particularly in advanced cases. This is particularly true for malignancies involving the maxillary sinus (whether arising from the oral mucosa or sinonasal mucosa), and those affecting both the upper nasal cavities and the nasopharynx (if originating from either site) [18].

Recently, significant attention has turned toward the potential role of the microbiome in influencing the development of head and neck SCC [19]. The human microbiome plays a crucial role in maintaining homeostasis within various body systems; it represents a diverse collection of microorganisms that coexist in symbiotic relationships within various human habitats. It is essential for supporting the immune system and overall health. Because of the unique nature of different microbial niches, the composition of these microorganisms varies in various anatomical sites, such as the airways, digestive system, and skin. The microbiota is affected by various factors, including environmental influences, the age and immune status of the host, and interactions within the microbiota itself. When the balance of the human microbial community is disrupted, beneficial and commensal bacteria that help control the overgrowth of harmful bacteria are often diminished [20].

In the nasal and sinonasal tract, a diverse and balanced microbial community is essential for immune function and mucosal barrier integrity [21]. However, perturbations to this balance, referred to as dysbiosis, have been implicated in various diseases, including allergic rhinitis (AR) and chronic rhinosinusitis (CRS), and now are suspected to contribute to malignancies. Recent evidence indicates that the composition and changes in the microbiome, as well as the presence of specific microbes in certain niches, can initiate tumor formation and promote tumor progression in vivo [22,23,24]. Furthermore, recent studies have unexpectedly revealed that microbiota play a key role in cancer development, primarily by impacting host cell growth and death, modifying immune system function, and influencing host metabolism [25,26].

Research specifically linking the microbiome to nasal malignancies is still limited. However, the general relationship between microbiota and head and neck cancers suggests that dysbiotic conditions may contribute to carcinogenesis in the nasal district [27]. Some authors reported that inflammation caused by bacterial imbalances might play a role in promoting the development of malignancies in these areas [28]. Further studies are needed to explore this potential connection. The aim of this paper is to review the emerging scientific evidence linking microbial dysbiosis in the nasal vestibule, nasal cavity, and sinonasal district with the development of SCCs. We explore the mechanisms through which an altered microbiome may contribute to carcinogenesis, focusing on chronic inflammation, immune evasion, and pathogen-associated molecular pathways.

We conducted a comprehensive search of all relevant papers across three major medical databases: PubMed, Scopus, and the Cochrane Library. We considered all available documents on the topic from the inception of each database up to September 2024. Additionally, we performed a manual search of the key literature from otolaryngology conferences and used citation chaining to ensure that no relevant articles were overlooked. The search utilized a combination of key terms: “Microbiota”, “Microbiome”, “Nasal”, “Nose vestibule”, “Carcinoma”, “Cancer”, and “Tumor”. The inclusion criteria focused on original articles, encompassing both prospective and retrospective studies, as well as review articles. Exclusions were made for single case reports, conference papers, articles not published in English, and letters to the editor.

## 2. The Nasal Microbiome: An Overview

The nasal district hosts a diverse range of microorganisms, including bacteria, fungi, and viruses. In healthy individuals, the nasal microbiota helps maintain mucosal integrity, modulates immune responses, and protects against colonization by pathogenic organisms. This is important because the upper airway is constantly exposed to airflow from the external environment. As such, the upper airways play essential physiological roles, including humidifying, warming, and filtering inhaled air. The nasal cavities connect to the external environment via the nasal vestibule, acting as a crucial transition zone that links the outside to the lower airways and the gastrointestinal system. Additionally, individuals inhale around 10^4^–10^6^ biological particles per cubic meter of air daily. Beyond these biological particles, the upper airways are subjected to physical and chemical weathering agents, such as humidity, oxygen, and various immunological or nutritional factors. These factors are significant as they contribute to the development of distinct microenvironments within different regions of the upper airway, including the nasal vestibule, nasal cavities, paranasal sinuses, nasopharynx, Eustachian tubes, middle ear cavities, oral cavity, oropharynx, and larynx. As a result, these different microenvironments in the upper airway host distinct microbial communities composed of both transient and resident microorganisms in differing ratios [29].

In the literature, the most commonly reported sampling sites for analyzing the upper airway microbiome are the nasal vestibule, middle meatus, and nasopharynx. The primary function of the nasal mucosa, which involves clearing inhaled air, may contribute to the high diversity observed in mucosal samples across these areas [30,31].

**Figure 1:**
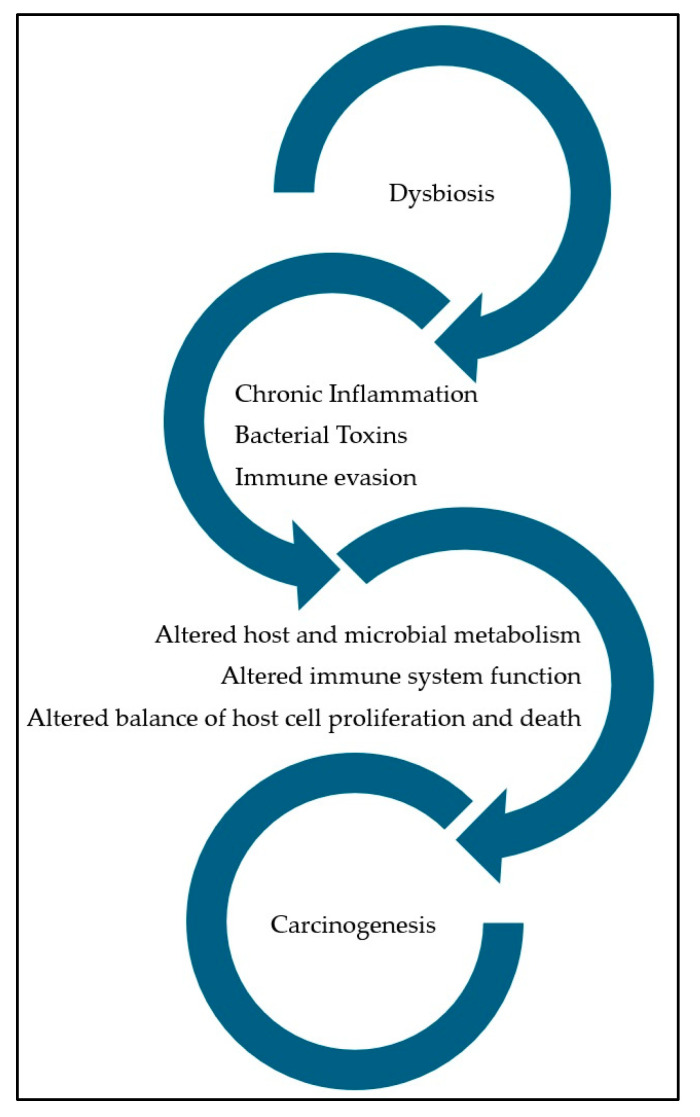
Potential implications of dysbiosis contributing to carcinogenesis.

The nasal vestibule surface, with its epithelium comprising sebaceous glands and vibrissae, is relatively drier compared to other regions of the upper airway and is the most exposed to the external environment. These vibrissae are able to trap larger particles (>3 μm) from inhaled air, while smaller particles, including microorganisms, are captured in a layer of mucus lining the nasal cavity. This mucus is then transported by ciliated epithelial cells from the nose to the esophagus through a process identified as mucociliary clearance [32,33]. The middle meatus is particularly significant for nasal microbiome research because it serves as the convergence point for secretions from the anterior part of the nasal cavity, anterior ethmoid, maxillary sinus, and frontal sinus [34]. The microbial community of the nasal vestibule is primarily dominated by species belonging to the genus *Corynebacterium*, *Staphylococcus*, *Propionibacterium*, and *Moraxella*. Notably, the nasal vestibule serves as a key reservoir for *Staphylococcus aureus* [35]. Regarding the sinonasal tract, this area includes rich diverse bacterial communities, with *Staphylococcus aureus*, *Staphylococcus epidermidis*, and *Propionibacterium acnes* representing the most abundant species [36]. The nasal microbiome usually correlates with the skin microbiome and, due to its lower complexity compared to, for instance, the oral microbiome, it is more susceptible to changes caused by environmental factors that can disrupt this delicate balance [37]. Such distruptions can lead to colonization [38] and proliferation of potentially opportunistic species that, by altering the immune homeostasis, may contribute to chronic inflammatory states, such as AR and CRS [39,40]. In patients with CRS, studies have reported a decrease in microbial diversity and an overrepresentation of certain bacteria, such as *Staphylococcus aureus* and *Pseudomonas aeruginosa* [41]. This imbalance is associated with prolonged inflammation and mucosal damage, which, over time, may increase the risk of developing malignancies [42] as described in Figure 1.

## 3. Mechanisms Linking Dysbiosis to Carcinogenesis

### 3.1. Chronic Inflammation

One of the primary mechanisms through which microbial dysbiosis can potentially contribute to nasal SCC is chronic inflammation [28].

In healthy individuals, the nasal mucosa is constantly exposed to environmental antigens and microorganisms. The nasal microbiome plays an essential role in modulating local immune responses and preventing excessive inflammation. However, colonization and overgrowth of bacteria capable of triggering and sustaining inflammatory responses may impair the local homeostasis, which is otherwise maintained by the nasal microbiome that modulates local immune responses to prevent excessive inflammation. In this regard, Gan et al. emphasized that the recurrence of chronic rhinosinusitis with nasal polyps (CRSwNP) after adequate endoscopic sinus surgery may potentially be associated with a state of dysbiosis, specifically characterized by a decreased presence of protective microorganisms and a higher prevalence of pathogenic microorganisms, including *Staphylococcus aureus* [43].

Chronic inflammation is a well-established risk factor for cancer in various tissues. Several authors report that long-term inflammation leads to the production of reactive oxygen species (ROS) and pro-inflammatory cytokines and chemokines, which can cause DNA damage, disrupt cellular processes, and promote malignant transformation. ROS have long been linked to cancer, with various tumor cells exhibiting elevated ROS levels compared to their normal counterparts. These increased ROS levels are believed to be oncogenic, leading to damage in DNA, proteins, and lipids, which in turn promotes genetic instability and tumorigenesis. Additionally, ROS function as signaling molecules in cancer, contributing to abnormal cell proliferation, metastasis, resistance to apoptosis, and angiogenesis, as well as causing differentiation blocks in certain cancer types. Elevated ROS levels create a pro-tumorigenic environment by activating pro-survival signaling pathways, impairing tumor suppressor gene function, enhancing glucose metabolism, enabling adaptations to hypoxia, and fostering the emergence of oncogenic mutations [44,45,46]. A further mechanism in bacterial carcinogenesis entails the activation of nuclear factor kappa B (NF-κB), which represents a crucial player in this process. Specifically, NF-κB is activated by several bacterial components, triggering the release of pro-inflammatory cytokines, which are related to cancer development [47]. Likewise, the activation of tumor necrosis factor alpha (TNF-α) and multiple inflammatory cytokines, including IL-6, IL-10, and IL-23, play a critical role in the cancer-promoting mechanisms associated with certain microbes [48].

Additionally, smoking can interfere with the balance of the nasal microbiome and increase the colonization by pathogenic bacteria [49,50,51]. It is widely acknowledged as a significant contributor to the development of nasal and sinonasal SCC, particularly in the malignant degeneration of sinonasal inverted papilloma [52]. Also, regarding the nasal vestibule, smoking is considered a risk factor for the development of SCC, as various studies in the literature show that the majority of patients with SCC are either smokers or former smokers [53].

Interestingly, Beachler et al. emphasized that chronic sinusitis may play a role in the development of some head and neck cancers, including nasopharyngeal cancer, HPV-related oropharyngeal cancer, and cancers of the nasal cavity and paranasal sinuses, potentially due to immunodeficiency or chronic inflammation [54].

### 3.2. Immune Evasion

An altered microbiome can significantly impair the local immune response, which may facilitate the persistence of potentially malignant cells within the tissue. In a healthy physiological state, the immune system plays a crucial role in actively surveilling and eliminating any abnormal or pre-cancerous cells that may arise. However, under conditions of dysbiosis, certain bacterial species may become predominant and produce various factors that can suppress immune function or promote mechanisms of immune evasion. This disruption in the balance of microbial communities can, therefore, compromise the immune system’s ability to effectively target and eliminate these potentially harmful cells [55]. For instance, *Staphylococcus aureus*, frequently overrepresented in dysbiotic nasal microbiomes, secretes superantigens and other virulence factors that can disrupt normal immune responses [56,57,58].

### 3.3. Direct Carcinogenic Effects of Bacterial Toxins

Certain bacterial species have the capacity to produce toxins and metabolites that directly promote carcinogenesis. For instance, chronic inflammation caused by ongoing *Staphylococcus aureus* infections can result in DNA damage, interfere with cellular signaling pathways, and contribute to the creation of an immunosuppressive microenvironment that promotes and supports cancer development. Furthermore, *Staphylococcus aureus* produces various toxins and metabolites that can interact directly with host cells, potentially leading to oncogenic changes. Indeed, chronic *Staphylococcus aureus* infections have been associated with an increased risk of skin cancer and oral cancer [59].

Another bacterium that can colonize the nasal mucosa is *Pseudomonas aeruginosa*, which produces important virulence factors, including pyocyanin, which triggers ROS production, causing oxidative stress in epithelial cells [60]. Over time, this oxidative stress can result in genetic mutations, genomic instability, and cellular transformation, paving the way for malignancy. In this regard, a *Pseudomonas* infection of the nose has been reported in some cases as an unusual complication of nasal surgery, or as an etiological agent of rhinosinusitis in immunocompromised patients [61,62].

A summary of the aforementioned mechanisms linking dysbiosis to carcinogenesis is presented in Table 1.

## 4. Fungal and Viral Contributions to Nasal Carcinogenesis

Fungi and viruses may play a crucial role in nasal carcinogenesis, either independently or through interactions with the bacterial microbiome.

### 4.1. Fungal Involvement

Fungal communities that inhabit our bodies are collectively known as the mycobiome. This mycobiome is frequently overlooked as a possible factor in disease development, mainly because it is less abundant (<0.1% of the total microbiota) and less diverse. Despite this, fungi are considerably larger than bacteria and possess metabolic gene clusters that align with various ecological requirements. Compared to the bacteriome, information about the mycobiome is limited. However, the recent application of advanced genomic sequencing techniques in fungal studies has enhanced our knowledge of their roles in health and disease. The mycobiome can be located in various anatomical regions including the oral cavity, airways, skin, vagina, and gastrointestinal tract [63].

In contrast to the bacterial microbiome, there has been a lack of studies investigating the sinus mycobiome in nasal inflammatory conditions; the most recent findings about this topic have highlighted that, like the bacterial microbiome, decreased fungal diversity may play a significant role in the mechanism of nasal dysbiosis.

Differences in mycobiome diversity between CRS patients and healthy individuals indicate that changes in the fungal mycobiome may contribute to disease pathogenesis in a manner similar to the bacterial microbiome [64].

Fungi, such as *Aspergillus* and *Candida*, have been implicated in the chronic inflammation associated with sinonasal diseases [65]. Chronic fungal infections that have persisted over long periods can significantly contribute to ongoing mucosal irritation and disrupt normal immune function, thereby creating an environment that may facilitate the development of malignancy.

### 4.2. Viral Involvement

Viruses are responsible for about 10% to 15% of all cancer cases globally. Various viruses have been implicated in the development of cancer, particularly several DNA viruses, which include Kaposi’s sarcoma herpesvirus (KSHV), Merkel cell polyomavirus (MCV), Human Papilloma Virus (HPV), Epstein–Barr virus (EBV), Hepatitis B virus (HBV), and Simian virus 40 (SV40). In addition, there are at least two RNA viruses involved in carcinogenesis: Human T-lymphotropic virus-1 (HTLV-1) and Hepatitis C virus (HCV). Notably, HPV and EBV are the oncogenic viruses most commonly linked to cancers of the head and neck region [66].

#### 4.2.1. Human Papilloma Virus (HPV)

The incidence of head and neck cancers related to HPV has been on the rise, as evidenced by the increasing rates of oropharyngeal squamous cell carcinoma (OPSCC) over the past few decades [67]. Recently, research has focused on the virus’s role in these cancers, with studies highlighting its prognostic significance in OPSCC [68]. Although HPV plays a clear etiological role in certain head and neck squamous cell carcinomas, the involvement of HPV in nasal malignancies is less clear. Recent studies have suggested that the presence of HPV in the sinonasal cavities, coupled with microbiome dysbiosis, may contribute to malignancy by allowing persistent viral infection [69,70]. Interestingly, it has been reported that up to 25% of malignancies arising in the sinonasal district contain transcriptionally active HPV [71]. Moreover, the virus is supposed to contribute to the transformation of sinonasal inverted papillomas into malignant carcinomas [72,73].

Furthermore, there are some reports in the literature of HPV being involved in the etiopathogenesis of nasal vestibule carcinoma. In this regard, Vital et al. examined p16 overexpression and high-risk human papilloma virus (HR-HPV) infection in nasal vestibule SCC, revealing a correlation between HR-HPV and p16 overexpression but without any influence on the outcome [74]. Yamamura et al. examined the presence of HPV-DNA using polymerase chain reaction (PCR) and assessed p16 status in five patients affected by nasal vestibule SCC. Three of these patients were treated with chemoradiation therapy, one with surgery, and one with surgery followed by radiation therapy. The authors found that four of the five cases were p16-positive, and one case was positive for HR-HPV infection [75]. Owusu-Ayim et al. described a 68-year-old man affected by SCC of the nasal vestibule. Immunohistochemistry revealed that the lesion was characterized by a surface epithelium exhibiting high-grade cytonuclear atypia and was positive for p16 and HPV infection [76].

#### 4.2.2. Epstein–Barr Virus (EBV)

Interestingly Epstein–Barr Virus (EBV) has been found in a significant proportion of sinonasal SCCs [77]. However, since a similar proportion of EBV infection was detected in nasal polyps, its effective role in sinonasal SCC carcinogenesis should be considered questionable [78]. Also, for the nasal vestibule, studies investigating the role of EBV in SCC are rare, and specifically, Paulino et al. found no association between nasal vestibule SCC and Epstein–Barr Virus infection [79].

## 5. Clinical Implications and Potential Therapeutic Approaches

### 5.1. Microbiome-Targeted Interventions

Probiotic therapies, designed to increase microbial diversity and promote the growth of beneficial commensal bacteria, are currently being explored in other cancers [80]. Further research is needed to understand whether this therapeutic strategy could potentially be applied to nasal malignancies.

Current research demonstrates the effectiveness of probiotics in the potential prevention of cancer and as an adjunctive treatment during anticancer chemotherapy. However, clinical trials remain insufficient to definitively confirm the potential of probiotic microorganisms in this context [81].

Upper respiratory tract probiotics, encompassing both traditional Lactobacillales and next-generation candidate probiotics (e.g., Dolosigranulum), may provide a natural support to standard treatment options. Although this area remains relatively underexplored compared to gut probiotic research, the application of probiotics in topical formulations is noteworthy. They have the potential to address various aspects of upper respiratory tract diseases due to their multifaceted mechanisms of action, which include microbiome restoration, antimicrobial activity, immunomodulation, and enhancement of barrier function [82].

### 5.2. Antibiotic and Antifungal Therapies

In cases where pathogenic bacteria or fungi are detected, targeted antibiotic or antifungal treatments may reduce inflammation and restore normal mucosal function. However, indiscriminate use of antibiotics could further disrupt the microbiome and increase the risk of developing prolonged inflammatory conditions such as CRS, underscoring the need for precision medicine approaches that take individual microbiome profiles into account [83].

### 5.3. Immunotherapy

Since dysbiosis is associated with immune dysfunction, combining microbiome modulation with immunotherapies may be a promising approach for treating nasal SCC in the advanced stage [84]. Enhancing the immune system’s ability to detect and eliminate malignant cells, while simultaneously restoring microbial balance, could improve clinical outcomes for patients.

Currently, immune checkpoint inhibitors (ICIs) are recognized as one of the first-line therapies for many unresectable solid tumors. However, evidence regarding the efficacy of ICIs in sinonasal malignancies is limited, and no ICIs have been approved for use in treating sinonasal SCC to date [85].

## 6. Future Directions for Research

The area of microbiome research specifically related to nasal malignancies is still in its very early stages of development, and a significant amount remains to be thoroughly understood and clarified. Future studies should aim to systematically characterize the microbiome in larger and more diverse cohorts of patients diagnosed with nasal vestibule or sinonasal SCC in order to expand the existing findings; carefully explore the causal relationships that exist between specific microbial species and the complex process of carcinogenesis by utilizing both animal models and in vitro experimental systems; and rigorously investigate the potential for microbiome-based biomarkers to aid in the early detection, diagnosis, and risk stratification for various types of nasal malignancies. Furthermore, additional research is also critically needed to explore the possible role of the mycobiome (the fungal microbiome) and the virome (the viral microbiome) in the intricate context of nasal carcinogenesis.

## 7. Conclusions

The relationship between microbial dysbiosis and nasal SCCs is an emerging area of scientific inquiry. Alterations in the nasal microbiome, characterized by decreased diversity and the overrepresentation of pathogenic microorganisms, appear to play a significant role in promoting chronic inflammation and immune evasion, and may promote carcinogenesis. While more research is needed to fully elucidate the mechanisms at play, understanding the role of the microbiome in nasal vestibular and non-vestibular SCCs could pave the way for novel therapeutic interventions aimed at preventing these aggressive malignancies.

## Figures and Tables

**Table 1:** Mechanisms linking dysbiosis to carcinogenesis.

Mechanisms Linking Dysbiosis to Carcinogenesis	Molecular Mediator	Molecular Mechanism	References
Chronic Inflammation	Reactive oxygen species	DNA damage Cellular process disruption Malignant transformation promotion	[44,45,46]
	Nuclear factor kappa B		[47]
	Tumor necrosis factor α		[48]
	Inflammatory cytokines		
Immune Evasion	Superantigens and other virulence factors secretion (*Staphylococcus aureus*)	Normal immune response disruption	[56,57,58]
Bacterial Toxins	Toxins and metabolites production (*Staphylococcus aureus*)	DNA damage Interference with cellular signaling Immunosuppressive environment	[59]
	Pyocyanin (*Pseudomonas aeruginosa*)	Reactive oxygen species triggering	[60]

## Data Availability

No new data were created or analyzed in this study. Data sharing is not applicable to this article.
